# Identification of disulfide cross-linked tau dimer responsible for tau propagation

**DOI:** 10.1038/srep15231

**Published:** 2015-10-15

**Authors:** Dohee Kim, Sungsu Lim, Md. Mamunul Haque, Nayeon Ryoo, Hyun Seok Hong, Hyewhon Rhim, Dong-Eun Lee, Young-Tae Chang, Jun-Seok Lee, Eunji Cheong, Dong Jin Kim, Yun Kyung Kim

**Affiliations:** 1 Korea Institute of Science and Technology (KIST), Brain Science Institute, Center for neuro-medicine, Seoul 136-791, South Korea; 2 Department of Biotechnology, Translational Research Center for Protein Function Control, College of Life Science and Biotechnology, Yonsei University, Seoul 120-749, South Korea; 3 Biological Chemistry, University of Science and Technology (UST), Daejon 305–333, South Korea; 4 Korea Institute of Science and Technology (KIST), Brain Science Institute, Center for Neuroscience, Seoul 136-791, South Korea; 5 Medifron-DBT Inc., Ansan, 425-839, South Korea; 6 Department of Neuroscience, University of Science and Technology (UST), Daejon 305–333, South Korea; 7 Advanced Radiation Technology Institute, Korea Atomic Energy Research Institute, Jeongeup 580-185, South Korea; 8 Department of Chemistry & Med Chem Program, National University of Singapore, 3 Science Drive 2, 117543 Singapore (Singapore); 9 Singapore BioImaging Consortium, Agency for Science, Technology and Research, 11 Biopolis Way, 138667 Singapore (Singapore); 10 Korea Institute of Science and Technology (KIST), Molecular Recognition Research Center, Seoul 136-791, South Korea

## Abstract

Recent evidence suggests that tau aggregates are not only neurotoxic, but also propagate in neurons acting as a seed for native tau aggregation. Prion-like tau transmission is now considered as an important pathogenic mechanism driving the progression of tau pathology in the brain. However, prion-like tau species have not been clearly characterized. To identify infectious tau conformers, here we prepared diverse tau aggregates and evaluated the effect on inducing intracellular tau-aggregation. Among tested, tau dimer containing P301L-mutation is identified as the most infectious form to induce tau pathology. Biochemical analysis reveals that P301L-tau dimer is covalently cross-linked with a disulfide bond. The relatively small and covalently cross-linked tau dimer induced tau pathology efficiently in primary neurons and also in tau-transgenic mice. So far, the importance of tau disulfide cross-linking has been overlooked in the study of tau pathology. Here our results suggested that tau disulfide cross-linking might play critical role in tau propagation by producing structurally stable and small tau conformers.

Tau is a neuron-specific microtubule-associated protein^1^. In a healthy neuron, tau stabilizes microtubules and promotes microtubule assemble^2^. When pathologically altered, tau dissociates from microtubules and become aggregated into insoluble filaments called neurofibrillary tangles (NFTs)^3^. The accumulation of tau inclusion is characteristic of multiple neurodegenerative disorders collectively called tauopathies, including Alzheimer’s disease (AD) and frontotemporal dementia (FTD)^4^. Accumulating evidence have demonstrated that tau aggregates are not only neurotoxic, but also propagate in neuron acting as a seed for native tau aggregation^5^,^6^,^7^,^8^,^9^. Initially, tau aggregates were thought to be released from dead or dying tangle-bearing neurons, and spread in the brain. More recent evidence suggests that neurons release tau as a free soluble form^10^ or as packed into vesicle such as exosome^11^. Then, secreted tau is taken up by neighboring cells initiating tau pathology^12^. Although the mechanism remains unclear, prion-like tau transmission is now recognized as a key pathological mechanism spreading tau pathology in the brain.

In 2009, tau transmission hypothesis was firstly demonstrated in mice by injecting brain lysates containing tau aggregates^6^. When injected into cortex region, the brain lysates induced tau pathology in non-symptomatic tau-transgenic mice. In addition, the tau lesions spread from the injection sites to the synpatically connected regions demonstrating tau propagation in a brain. During the past years, diverse efforts have been made to characterize prion-like tau species. A number of post-translational modifications have been analyzed to characterize pathological tau modifications that are critical for transmission^9^,^13^. However, it is not easy to obtain a global picture of tau modifications since a number of different aspects were analyzed in various systems^14^. More recent evidence showed that tau pathology could be induced by synthetic tau fibrils without bearing any pathological modifications. Seeding of synthetic tau fibrils efficiently converts endogenous tau into pathological aggregates in cultured cells and tau-transgenic mice^7^,^8^,^9^. The studies imply that prion-like conformation, rather than a specific modification, might be critical to evoke tau transmission. However, the prion-like tau conformation inducing intracellular tau aggregation is controversial. In this study, we focused on characterizing the smallest tau conformation inducing intracellular tau aggregation.

To evaluate prion-like activity of tau, a reliable system for monitoring intracellular tau aggregation is necessary. To visualize tau aggregation in living cells, we recently developed a cell-based sensor, named tau-BiFC (bimolecular fluorescence complementation)^15^. In tau-BiFC system, non-fluorescent N- and C-terminal compartments of Venus protein are fused to tau, and Venus fluorescence turns on only when tau assembles together. By eliminating the background noise from monomeric tau, we could monitor and quantify intracellular tau-tau interaction from the early stage of aggregation. Diverse tau aggregates were prepared and the prion-like activity was measured and compared using tau-BiFC sensor.

## Results

### K18-P301L induced intracellular tau aggregation

**Figure 1:**
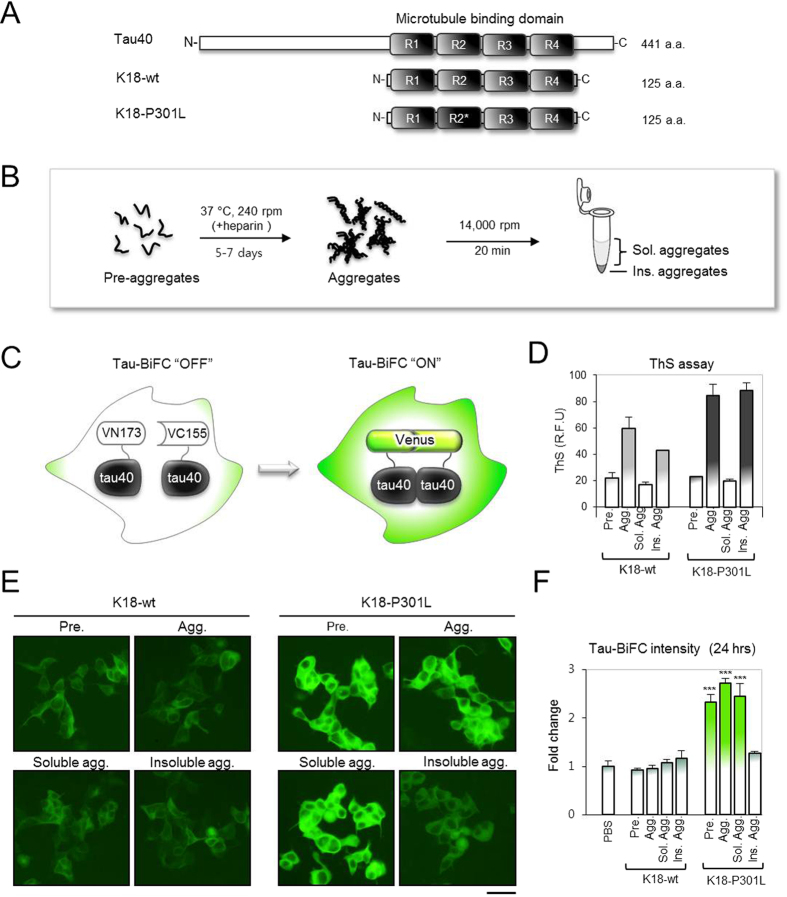
Evaluation of prion-like activity of tau aggregates. (**A**) Tau40 is a full-length human tau and K18 is a microtubule-binding domain containing four repeated regions (R1-R4). K18-P301L contains a point mutation in the R2 region. (**B**) A schematic diagram indicates preparation of tau aggregates (**C**) Tau-BiFC fluorescence turns on only when tau assembles together. (**D**) Thioflavin S (ThS) assay indicates the relative level of tau aggregation. Error bar represents S.D. of triplicate experiments. (**E**) To identify prion-like tau aggregates, K18-wt or K18-P301L aggregates (10 μg/mL) were treated to Tau-BiFC cells for 24 hrs. Then, Tau-BiFC cells were imaged by using Operetta® High Content Imaging System. Scale bar = 50 μm. (**F**) The intensity of BiFC fluorescence was quantified by using Harmony^TM^ software. Error bar indicates S.D. of triplicate experiments. Pre, pre-aggregates; Agg, aggregates; Sol, soluble; Ins, insoluble.

Exogenous tau aggregates were prepared by using a truncated tau fragment, K18 (Fig. 1A). During NFT formation, proteolysis occurs to remove the soluble N- and C-terminal region of tau. The remaining region bearing microtubule-binding domain is known to be responsible for tau aggregation and propagation^16^,^17^. Together with the wild-type K18 (K18-wt), K18 bearing a P301L mutation was also prepared. P301L mutation is associated with familial tauopathies known for frontotemporal dementia and parkinsonism linked to chromosome 17 (FTDP-17). It is known that P301L mutation reduces tau’s binding affinity to microtubules and increases the aggregation propensity^18^. Due to the spontaneous assemble and disassemble of K18, we considered the purified K18 fraction as a pre-aggregate state (Fig. S1). K18 aggregation was induced by the addition of dithiothreitol (DTT) and heparin. After 7 days, tau aggregation was evaluated with thioflavin S (ThS) assay^19^ and transmission electron microscopy (TEM) analysis^20^. ThS assay indicates the amount of β-sheet aggregates in the mixture and TEM analysis evaluates tau filament formation. In cases of pre-aggregate condition, K18-wt and K18-P301L exist as a soluble mixture neither showing ThS response nor noticeable aggregation on TEM images. In the aggregate condition, highly ordered-fibrillary structures were observed in both of K18-wt and K18-P301L aggregation mixture (Fig. S2). ThS assay indicated that K18-P301L has higher propensity to form β-sheet aggregates than K18-wt does (Fig. 1D).

To evaluate the prion-like activity of K18-wild type and K18-P301L, each of the pre-aggregates and aggregates were treated to the medium of tau-BiFC cells (Fig. 1C). In case of K18-P301L, both pre-aggregates and the aggregates induced tau-BiFC response dramatically by showing 2.1-fold and 2.5-fold increase respectively (Fig. 1F). K18-wt, regardless of its aggregation status, did not induce tau BiFC fluorescence response up to 24 hrs (Fig. 1E).

To specify further the infectious forms of K18-P301L, we divided the aggregates into soluble and insoluble fractions by centrifugation (Fig. 1B). The soluble and the insoluble fractions were evaluated by ThS assay (Fig. 1D). When treated to tau-BiFC cells, only the soluble fraction induced intracellular tau aggregation by showing 2.2-fold increase of tau BiFC fluorescence (Fig. 1F). Insoluble fraction that shows a strong ThS response did not induce any noticeable change of intracellular tau. This suggests that the transmittable species might be soluble oligomers that predominantly exist in the pre-aggregates and the soluble aggregates of K18-P301L.

### K18-P301L prefers to form disulfide cross-linked dimer

**Figure 2:**
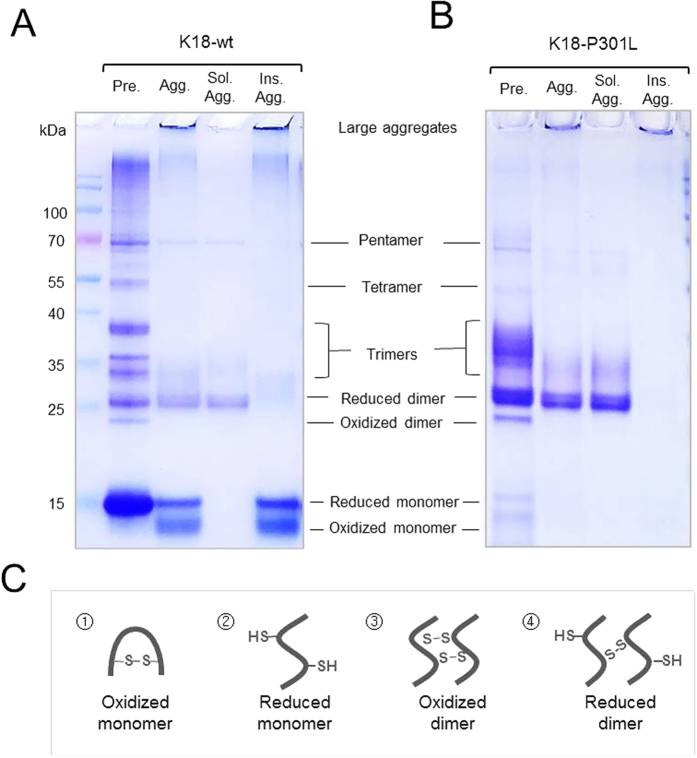
Separation of low molecular weight oligomers. Non-reducing SDS-PAGE (10–14%) visualizes disulfide cross-linked oligomers of (**A**) K18-wt and (**B**) K18-P301L. (**C**) Illustration of possible molecular species of tau monomers and dimers. Pre, pre-aggregates; Agg, aggregates; Sol, soluble; Ins, insoluble.

To analyze soluble tau aggregates, non-reducing polyacrylamide gel electrophoresis (PAGE) analysis was followed. Tau contains two cysteine residues that can form both intra- and inter-molecular disulfide bonds and the disulfide cross-linked oligomers can be visualized on a non-reducing SDS-PAGE gel. Although large aggregates are not separable on an SDS-PAGE gel, soluble oligomers ranging from monomer to pentamer are distinguishable on a gel^19^. In pre-aggregates condition, K18-wt showed a concentrated band of monomer with multiple bands of oligomers (Fig. 2A). In contrast, K18-P301L showed a concentrated band of a reduced dimer and trimers in the pre-aggregate condition (Fig. 2B). K18 fragment is able to form two types of dimers according to the number of disulfide bonds^19^,^21^. Due to the compact conformation, an oxidized dimer is expected to run faster on a PAGE-gel compared with the reduced dimer. The differential mobility of K18 dimers was confirmed by using K18-C291S mutant (Fig. S3). K18-C291S mutant contains only one cysteine residue; therefore, it forms only a reduced dimer. When compared, the reduced dimer of the C291S mutant was perfectly matched to the upper band of wild-type dimers. In case of K18-C291S/C322S mutant containing no cysteine residue, dimers and oligomers were not detectable on the non-reducing SDS-PAGE gel, supporting the importance of disulfide bridge in the formation of tau oligomers.

Interestingly, a strong band of dimer was observed both in the pre-aggregates and the soluble aggregate fraction of K18-P301L (Fig. 2B). Considering the comparable tau-BiFC responses of the pre-aggregates and soluble aggregates, the reduced dimer might be the responsible form to inducing intracellular tau aggregation. A band of reduced dimer was also observed in the pre-aggregates and the aggregates of K18-wt (Fig. 2A). However, the dimer band was quite faint compared to that of K18-P301L. The majority of K18-wt exists as a reduced monomer in pre-aggregate condition. Interestingly, monomer bands were appeared in the fraction of insoluble aggregates, not of soluble aggregates. This suggests that K18-wt aggregates are easily breakable into monomers. In comparison, K18-P301L aggregates were stable without generating monomers during preparation.

### Tau pathogenesis induced by K18-P301L dimer

**Figure 3:**
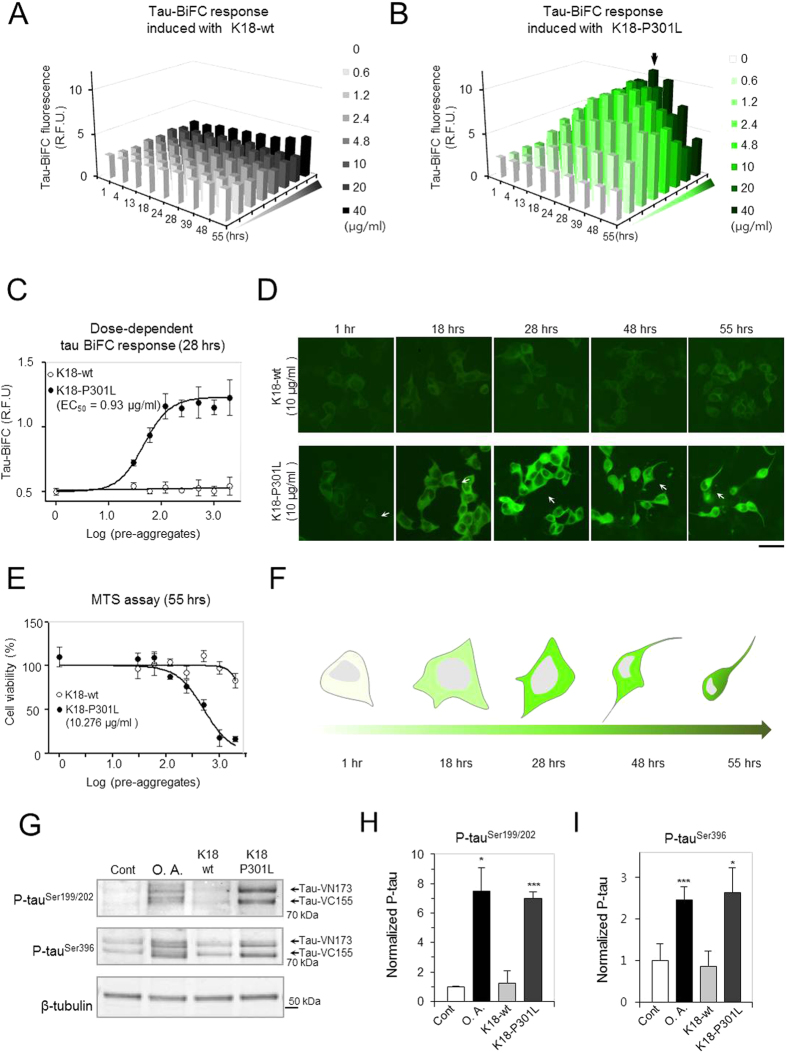
Dose-dependent effects of disulfide cross-linked tau dimers on tau transmission. (**A**,**B**) Tau-BiFC cells were incubated with various concentrations of K18-wt or K18-P301L dimers. Then, cellular responses of tau-BiFC fluorescence were imaged at various time points. Each data point of the graphs shown represents the mean of triplicate experiments (R.F.U.). The black arrow indicates the saturated response of tau-BiFC fluorescence at 28 hrs. (**C**) Dose-response curves of tau BiFC-fluorescence induced with K18-wt or K18-P301L at 28 hrs. Error bars indicate S.D. of triplicate experiments. The EC_50_ value was determined by Prism’s nonlinear regression analysis. (**D**) The fluorescence images present time-dependent changes of tau-BiFC cells in the presence of K18-wt or K18-P301L (10 μg/ml). Scale bar = 50 μm. (**E**) MTS assay indicates the cytotoxicity induced by the treatment of K18-wt and K18-P301L dimers. Error bar indicates S.D. of triplicate experiments. The IC_50_ value was determined by Prism’s nonlinear regression analysis. (**F**) Illustration indicates the morphological changes of a tau-BiFC cell in the presence of K18-P301L dimer. (**G**) For the immune-blot assay, tau-BiFC cells were incubated with 20 μg/ml of K18-wt or K18-P301L for 24 hrs. 30 nM of Okadaic acid (O.A.) was used as a positive control. Black arrows indicate full-length tau tagged with VN173 or VC155. (**H**,**I**) The relative amounts of phosphorylated tau were quantified and normalized with that of β-tubulin. Error bars represent S.D. from three independent experiments. The significance of the experiments was determined with paired t-test. *p < 0.05, ***p < 0.001.

To investigate the prion-like effect of disulfide cross-linked dimer, the soluble fractions of K18-wt and K18-P301L from aggregates were applied to the tau-BiFC cells at different doses, and the BiFC fluorescence change was monitored over time (Fig. 3A,B). Again, the K18-wt did not induce noticeable change of intracellular tau aggregation. Only after 55 hrs, tau-BiFC intensity slightly increased at the highest concentration (40 μg/ml) (Fig. 3A). This result implies that K18-wt dimer might be transmittable; however, the transmission capacity was much weaker than that of K18-P301L dimer. In contrast, K18-P301L dimer forcefully induced tau-BiFC fluorescence dose-dependently and also time-dependently (Fig. 3B). At high doses, tau-BiFC fluorescence intensities increased rapidly and become saturated at 28 hrs. The saturation point is marked as a black arrow in Fig. 3B. At 28 hrs, the half maximal effective concentration (EC_50_) of K18-P301L was 0.9 μg/ml (Fig. 3C).

Interestingly, the saturated BiFC fluorescence gradually decreased after 28 hrs. Morphological changes were observed in tau-BiFC cells treated with K18-P301L dimer (Fig. 3D). After reaching to the saturation time, the cell bodies become shrinked resulting in cell death. Representative cells from each time point were marked and illustrated in Fig. 3F. To evaluate tau-mediated cell death, MTS analysis was performed. At 55 hrs of incubation, cell viability decreased greatly upon the treatment of K18-P301L soluble fraction. The half maximal inhibitory concentration (IC_50_) of K18-P301L soluble fraction was 10.3 μg/ml (Fig. 3E). Although high molecular weight oligomers also exist in the mixture, the reduced dimer is the predominant species in the soluble fraction of K18-P301L (Fig. 2). To demonstrate the role of disulfide bridging in inducing intracellular tau aggregation, K18-P301L was pre-incubated with extremely high concentration of DTT (1 mM) (Fig. S8). Under strong reducing condition, most of K18-P301L dimers were monomerized. When treated to tau-BiFC cells, the rate of tau-BiFC maturation was lower than that of K18-P301L dimers. This result supports the importance of K18-P301L dimer in inducing intracellular tau aggregation. Again, our results strongly suggest that K18-P301L dimer is the smallest tau conformation initiating intracellular tau pathogenesis including tau aggregation, phosphorylation and tau-mediated cytotoxicity.

Next, immunoblot assay was followed to evaluate the level of intracellular tau phosphorylation. Tau-BiFC cells were incubated with 20 μg/ml of K18-wt or K18-P301L soluble fractions for 24 hrs. Okadaic acid, a protein phosphatase 2A inhibitor, was treated as a positive control. An immunoblot assay with anti-phospho tau antibodies shows two tau constructs conjugated with VN173 or VC155. Upon the treatment of K18-P301L dimer, tau phosphorylation was increased 7.0-fold at Ser199/202 (Fig. 3H) and 2.6-fold at Ser396 (Fig. 3I), of which phosphorylation levels were comparable to that of okadiaic acid.

There are two possible factors that contribute the superior prion-like activity of K18-P301L dimer; the first factor is the rate of internalization and the second factor is the seeding efficiency. To investigate the rate of internalization, N-terminal of K18-wt and K18-P301L were labeled with Cy5. Then, the labeled K18-wt and K18-P301L were treated to tau-BiFC cells or primary neurons (Fig. S4). Fluorescence microscopy images showed that there was no significant difference on cellular uptake of K18-wt and K18-P301L. This indicates that the rate of internalization might not be the factor that leads the prion-like activity of K18-P301L dimer. Next, we compared the seeding efficiency facilitating the aggregation of full-length tau *in vitro* (Fig. S5). Toward that, tau-BiFC cell lysates were prepared and incubated with K18-wt or K18-P301L. After 2 days, only K18-P301L-treated cell lysates showed increased BiFC-fluorescence. Our results clearly demonstrated that superior seeding efficiency of K18-P301L dimer is the main factor that induce intracellular tau aggregation.

### K18-P301L dimer induced neuronal degeneration

**Figure 4:**
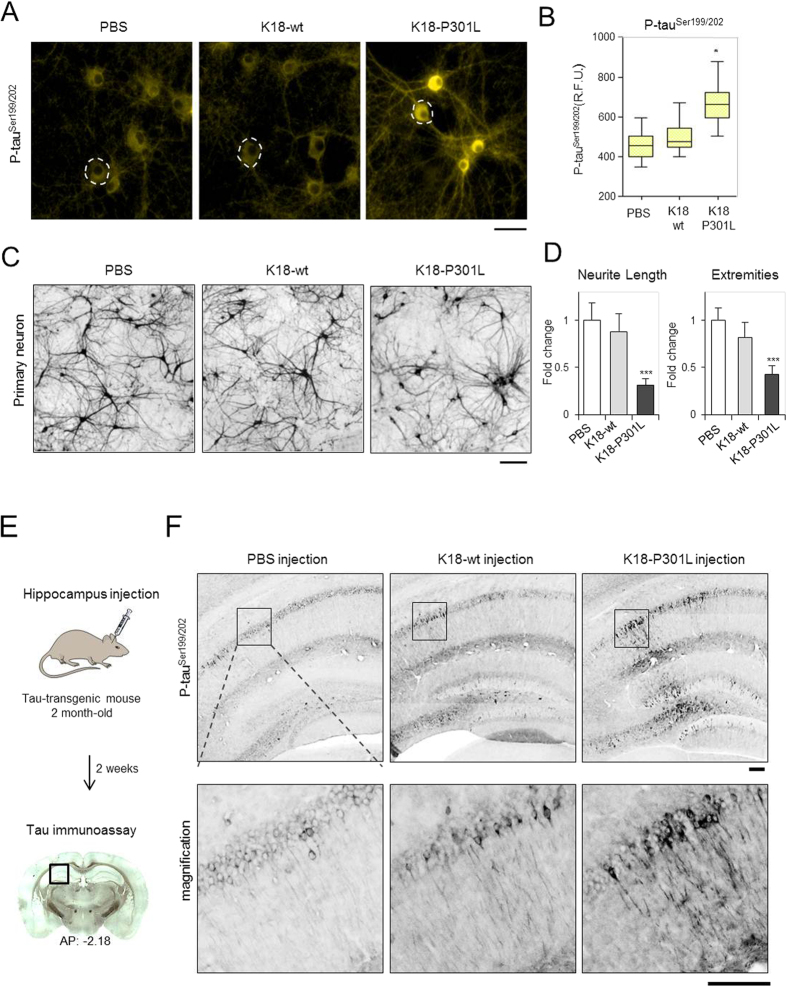
Tau transmission induced by K18-P301L dimer. (**A**) Primary neurons were treated with PBS, K18-wt or K18-P301L for 24 hrs. Then, neurons were stained with anti-phospho tau^Ser199/Ser202^ antibody. Scale bar = 50 μm. (**B**) The box plot graph indicates the distribution of cellular phospho-tau intensities. The boxes represent 25% to 75% range of data. (**C**) Neuronal degeneration induced by K18-P301L dimer. Primary neurons were incubated with K18-wt and K18-P301L for 48 hrs and stained with NeuO, a neuron selective probe (**D**) Quantification of the length of neurites and the number of extremities. Error bars indicate S.D. from three independent experiments. ***p < 0.001. (**E**) The schematic diagram, drawn by D. Kim using ChemBioDraw 13.0. software, represents the hippocampal injection of K18-wt or K18-P301L into the pre-symptomatic tau transgenic mice (MAPT^P301L^). PBS was injected as a control. Two weeks after the injection, brains were extracted and dissected for further analysis. (**F**) The representative images of the brain slices stained with anti-phospho tau^Ser199/Ser202^ antibody. The lower panels are the high-magnification images of the selected regions. Scale bar = 100 μm.

We further validated the prion-like activity of K18-P301L dimer in primary neurons. Primary hippocampal neurons were isolated from day-18 rat embryos. After 7 days of *in vitro* culture, K18-wt and K18-P301L dimers (10 μg/ml) were treated to the hippocampal neurons after 7 days of *in vitro* culture. After 48 hrs, neurons were fixed and immunofluorescence-stained with phospho-tau antibody (pSer199/202). The phosphorylated tau condensed in soma was quantified by using Harmony 3.1 software (PerkinElmer™). The box-plot represents the full range distribution of the immunofluorescence intensities of 50 ~ 60 neurons (Fig. 4B). As a result, endogenous tau phosphorylation was increased 1.5 fold in primary neurons with the treatment of K18-P301L dimer (Fig. 4A). In contrary, K18-wt dimer did not induce any significant change in primary neurons.

Next, to evaluate neuronal integrity, primary neurons were incubated with K18-wt or K18-P301L dimer. After 48 hrs, primary neurons were stained with NeuO, a neuron selective fluorescence probe (Fig. 4C)^22^. Then, fluorescence images were acquired and analyzed to evaluate the number of extremities and the length of neurites (Fig. S9). Neuronal degeneration was observed in the neurons treated with K18-P301L by showing 42% shortened neurites compared with PBS-treated neurons (Fig. 4D). The number of extremities was also significantly reduced in the neurons treated with K18-P301L (Fig. 4D). In contrary, K18-wt did not induce significant effect on the integrity of the primary neurons. These results clearly indicate that K18-P301L dimer activates tau pathogenesis not only in tau-BiFC model system, but also in primary neurons.

### K18-P301L dimer induced tau pathology in tau transgenic mice

Lastly, we investigated the prion-like activity of K18-P301L dimer in mouse brains. K18-wt and K18-P301L dimers were directly injected into hippocampus of 2-month-old tau transgenic mice (MAPT*P301L mice) expressing human full-length tau bearing P301L mutation (Fig. 4E). 2-month-old MAPT mice are considered as pre-symptomatic mice since MAPT mice are known to develop tau pathology after 5-months-old^23^. After 2 weeks of the injection, mice were perfused and brain tissues were prepared. For immunofluorescence analysis, brain slices near the injection site were selected (AP: −2.18), and stained with phospho-tau antibody (pSer199/202). Upon the injection of K18-P301L dimer, obvious tau phosphorylation was observed in hippocampal neurons (Fig. 4F). K18-wt dimer also slightly induced tau phosphorylation in hippocampal neurons. However, the level of phosphorylation was not significant as that of K18-P301L. GFAP (Glial fibrillary acidic protein) stains indicate the comparable brain damages caused by stereotaxic injection (Fig. S7). This result clearly demonstrates the prion-like activity of K18-P301L dimer in mouse brains.

To evaluate tau propagation in the brain, brain slices featuring dentate gyrus was stained with phospho-tau antibody (pSer 199/202). The selected brain slices (AP: −3.06) is far from the injection site (AP: −2.18), and also the anatomical connection between dentate gyrus and hippocampus has been known^24^. Although faintly stained, neurons bearing, hyperphosphorylated tau, were observed in the dentate gyrus of K18-P301L-injected mouse brain as an indication of tau propagation (Fig. S6). Our results clearly demonstrated that the induced tau pathology by K18-P301L dimer can be transmittable to the connected brain region.

## Discussion

Accumulating evidence have shows that tau aggregates propagate in neurons acting like a prion^5^,^6^,^7^,^8^,^9^. Pathogenic tau aggregates are released from affected neurons and propagate into unaffected neurons imposing their anomalous structure on benign tau molecules^11^,^25^. Hence, identifying the infectious templates of tau is of great interest. To act as a prion, (i) keeping a certain conformation is necessary to serve as a corruptive template for tau aggregation and also (ii) small size might be preferred for neuronal uptake. In this regards, low molecular weight oligomers and short filaments are considered as prion-like tau species^12^. Recently, Mandelkow and Klenerman’s group reported that K18 monomer bearing ΔK280 mutation is the smallest form that can induce *in vitro* tau aggregation^26^. However, they also indicated that the monomer was not able to induce neuronal toxicity. This suggests the importance of forming prion-like structure for tau transmission. Here, our data clearly suggest that disulfide cross-linked tau dimer is the smallest form to induce tau aggregation and intracellular propagation.

Then, an important question remains; whether the dimeric tau is actually secreted and circulating in the brain extracellular space. Although the secretion mechanism is debatable, the presence of tau dimers and trimers has been observed in the cerebrospinal fluid from AD patients^11^. We expect that the circulating tau dimers and trimers would actively transmit tau pathology in the patient’s brain.

The importance of tau disulfide bond formation has been neglected in the field of tau pathogenesis. However, oxidative stress is one of the key factors contributing to tau pathology^27^ and it is highly possible that disruption of cellular redox potential alters the oxidation state of tau, generating disulfide cross-linked tau dimers. The importance of tau disulfide bond formation has been neglected in the field of tau pathogenesis. Our results suggest that disulfide cross-linked tau dimer would play critical role in transmitting tau pathology in the brain.

## Methods

### Preparation of tau aggregate species

The DNA sequence coding K18 was cloned from full-length human tau (htau40) and inserted into a pET vector. K18 mutants (K18-P301L, K18-C291S and K18-C291S/C322S) were prepared by using the QuikChange site-directed mutagenesis kit (Stratagene). K18-wild type and K18-P301L mutant proteins were expressed and purified from E. *coli* BL21 (DE3). To induce tau aggregation, purified K18-wt and K18-P301L (35 μM) in phosphate-buffered saline (PBS, pH 7.4) were incubated with 0.1 mg/mL heparin (Sigma; MW 18 kDa) and 100 μM dithiothreitol (DTT) (Sigma) at 37 °C for 5–7 days^19^. To separate soluble and insoluble aggregates from aggregates, aggregates mixutre was centrifuged at 14,000 rpm for 20 min. Soluble aggregates (supernatant) were transferred to another tube and insoluble aggregates (pellet) were diluted with autoclaved PBS. Quantitative aggregation of K18-wt or K18-P301L was evaluated by a thioflavin S (ThS) fluorescence assay. Each state (pre-aggregates, aggregates, soluble aggregates or insoluble aggregates) of K18 mixture (5 μL) was transferred to a black 384-well plate with 45 μL of PBS containing 10 μM ThS (Sigma). ThS signal was measured at excitation wavelength of 430 nm and emission wavelength of 480–610 nm in a Flexstation2 spectrophotometer (Molecular Devices). Quantification data was analyzed by Student’s t-test with 95% significance level in Excel.

### Non-reducing SDS-PAGE analysis

Diverse K18-wt or K18-P301L aggregates was mixed with Laemmli sample buffer (Biorad Laboratories) without β-mercaptoethanol and resolved on SDS-PAGE (10–14%) gel. The gel was stained with Coomassie brilliant blue solution to visualize monomer to aggregates of K18-wt and K18-P301L.

### Tau-BiFC cell culture and K18 treatment

HEK293 Tau-BiFC cells were maintained in Dulbecco’s modified eagle medium (DMEM) supplemented with 10% fetal bovine serum (FBS), 10000 units/ml penicillin, 10000 μg/ml streptomycin and 1 μg/ml Geneticin (G418) in humidified atmosphere containing 5% CO2 at 37 °C. For exogenous K18 treatment, tau-BiFC cells were plated in a black transparent 384-well under the starvation medium (DMEM, 6% FBS, penicillin/streptomycine, G418). The next day, tau-BiFC cells were treated with K18-wt or K18-P301L at various concentrations. After the incubation, the fluorescence response in tau-BiFC cells was automatically imaged by Operetta (PerkinElmer™). The intensities of tau-BiFC fluorescence were analyzed using Harmony 3.1 software (PerkinElmer™). Quantification data was analyzed by Student’s t-test with 95% significance level.

### Immunoblot analysis

To quantify phosphorylation level of tau-BiFC cells, immunoblot assay was performed after 24 hrs of K18-wt or K18-P301L treatment. Total cell lysates were prepared by using CelLytic M (Sigma) containing protease and phosphatase inhibitor cocktail (Sigma). 15 μg of cell lysates were separated on SDS-PAGE gel (10%) and transferred to PVDF membrane. The level of tau phosphorylation was detected by immunoblot with phospho-tau antibody Ser199/202 (1:1000, abcam) and Ser396 (1:1000, abcam). anti-β tubulin antibody was used as a loading control. Quantification data was analyzed by Student’s t-test with 95% significance level.

### Primary neuron culture

All animal experiments were approved by the Korea Institute of Science and Technology, and the experimental protocols were carried out in accordance with the approved guidelines by Institutional Animal Care and Use Committee of Korea Institute of Science and Technology.

Primary neurons cultures were prepared from rat embryo (E18). Isolated hippocampus was washed with Hank’s Balanced Salt Solution (HBSS), followed with trypsin incubation for dissociation of cells. After trituration and centrifugation, supernatant was removed. Cells were resuspended with neurobasal medium (Gibco) with 2% B27 supplement (Gibco), 0.5 mM glutamax (Gibco), 100 units/ml penicillin, 100 μg/ml streptomycin, and 5% FBS. Resuspended cells were plated at 7.5 × 10^3^/well in poly-D-lysine coated 96 well plates. Cultured neurons were grown in 5% CO2 and 85% humidity in the above medium. After three days, the medium was changed to medium without FBS. The medium was replaced half/half every 3 days. K18-wt or K18-P301L was treated to primary neurons at 7 days *in vitro*.

### Immunofluorescence analysis

To detect endogenous tau phosphorylation in primary neurons, therneurons were fixed by 3.7% paraformaldehyde (PFA, Sigma) after 48 hrs of K18-wt and K18-P301L treatment. Then, the neurons were incubated in 0.1% triton-X in PBS for permeabilization. After washing with PBS, primary neurons underwent blocking step by 4% BSA for 1 hr followed with incubated with primary phospho-tau antibody Ser 199/202 (1:1000, abcam) overnight at 4 °C. Next day, primary neurons were stained with Alexa Fluor 488 or 633-conjugated secondary antibodies (1:1000, abcam). Images were obtained by the Operetta (PerkinElmer™). The fluorescence intensities were measured in 50 ~ 60 neuronal somas in 5 images from each PBS, K18-wt and K18-P301L-treated primary neurons. Quantification data was analyzed by F-test.

For neuronal degeneration analysis, primary neurons were stained with 250 nM NeuO, neuron selective probe^22^. After 1 hr incubation with NeuO, images were obtained by the Operetta (PerkinElmer™,). The neurite length and the number of extremities were analyzed by Harmony 3.1 software (PerkinElmer™) (Fig. S9). Quantification data was analyzed by Student’s t-test with 95% significance level.

### Stereotaxic surgical injection

MAPT transgenic mice were used for this experiment. The mice expressed the human P301L mutation of the microtubule-associated protein tau gene (MAPT)^23^. For induction of endogenous tau phosphorylation *in vivo*, either K18-wt or K18-P301L was injected into 2 months old of MAPT transgenic mice. Before injection, K18-wt and K18-P301L were diluted with PBS at 1.3 mg/ml. The mice were anesthetized with avertin (200 mg/kg, i.p.) and then placed in a stereotaxic device. The target site is a hippocampus at AP: −1.8 mm, ML: ±2.0 mm and DV: −2.0 mm based on the atlas of Paxinos and Franklin^28^. A hole was made at AP: −1.8 mm, ML: ±2.0 mm in a scalp for needle injection. A 27 gauge dental needle connecting with 25 μL Hamilton syringe was inserted at DV: −2.2 mm and stayed for 30 min. Then, the needle was moved up about 200 μm and either 5.2 μg of K18-wt or K18-P301L was injected by pressure injection at 0.15 μ ∙ L-1. As a control, 4 μL of PBS was injected. After injection, the needle was left for 20 min more to diffuse K18-wt and K18-P301L in the target region. Then, the needle was removed and skin was sutured.

### Histology and immunofluorescence analysis

Two weeks after injection of K18-wt and K18-P301L, the mice were transcardially perfused with PBS and then, fixed with PBS containing 4% PFA of pH 7.2 ~ 7.4. The brains were removed and kept in 4% PFA overnight and 30% sucrose subsequently until brains were sunk. Following fixation, the brains were embedded with O.C.T. (Tissue-TEK). The brains were cut coronally using cryotome at 30 μm thickness. To detect endogenous tau phosphorylation and evaluate immune response derived by injection, brain slices were fixed by 3.7% PFA, followed with incubated in 0.1% PBS-T for permeabilization. After washing with PBS, brain slices underwent blocking step by 4% BSA for 1 hr and then, incubated primary antibody overnight at 4 °C; pSer199/202 (1:200, abcam) and GFAP (1:500, DAKO). Next day, brain slices were stained by Alexa Fluor 488 or 633-conjugated secondary antibodies (1:500, abcam). All images were taken by Operetta (PerkinElmer™, USA).

## Additional Information

**How to cite this article**: Kim, D. *et al.* Identification of disulfide cross-linked tau dimer responsible for tau propagation. *Sci. Rep.* **5**, 15231; doi: 10.1038/srep15231 (2015).

## Supplementary Material

Supplementary Information
