# Supplementary material for: Identification of disulfide cross-linked tau dimer responsible for tau propagation
Source: Sci Rep. 2015 Oct 15;5:15231. doi: 10.1038/srep15231 (PMC4606741; doi:10.1038/srep15231)
Supplement: Supplementary Information [file srep15231-s1.pdf]

Identification of disulfide cross-linked tau dimer responsible for tau propagation

Dohee Kim[a,b†], Sungsu Lim[a†], Md. Mamunul Haque[a,c], Nayeon Ryoo[d], Hyun Seok Hong[e], Hyewhon Rhim[d,f], Dong-Eun Lee[g], Young-Tae Chang[h,i], Jun-Seok Lee [j,c], Eunji Cheong[b], Dong-jin Kim[a], and Yun Kyung Kim[a,c*]

[a]*Korea Institute of Science and Technology (KIST), Brain Science Institute, Center for neuro-medicine, Seoul 136-791, South Korea*

[b]*Department of Biotechnology, Translational Research Center for Protein Function Control, College of Life Science and Biotechnology, Yonsei University, Seoul 120-749, South Korea*

[c]*Biological Chemistry, University of Science and Technology (UST), Daejon 305–333, South Korea*

[d]*Korea Institute of Science and Technology (KIST), Brain Science Institute, Center for Neuroscience, Seoul 136-791, South Korea*

[e]*Department of Biochemistry and Biomedical Sciences; College of Medicine; Seoul National University; Seoul, Korea ; Medifron-DBT; Ansan,South Korea*

[f]*Department of Neuroscience, University of Science and Technology (UST), Daejon 305–333, South Korea*

[g]*Advanced Radiation Technology Institute, Korea Atomic Energy Research Institute, Jeongeup 580-185, South Korea*

[h]*Department of Chemistry & Med Chem Program, National University of Singapore, 3 Science Drive 2, 117543 Singapore (Singapore)*

[i]*Singapore BioImaging Consortium, Agency for Science, Technology and Research, 11 Biopolis Way, 138667 Singapore (Singapore)*

[j]*Korea Institute of Science and Technology (KIST), Molecular Recognition Research Center, Seoul 136-791, South Korea*

Contents

Supplementary Figure 1

Supplementary Figure 2

Supplementary Figure 3

Supplementary Figure 4

Supplementary Figure 5

Supplementary Figure 6

Supplementary Figure 7

Supplementary Figure 8

Supplementary Figure 9

Supplementary Methods

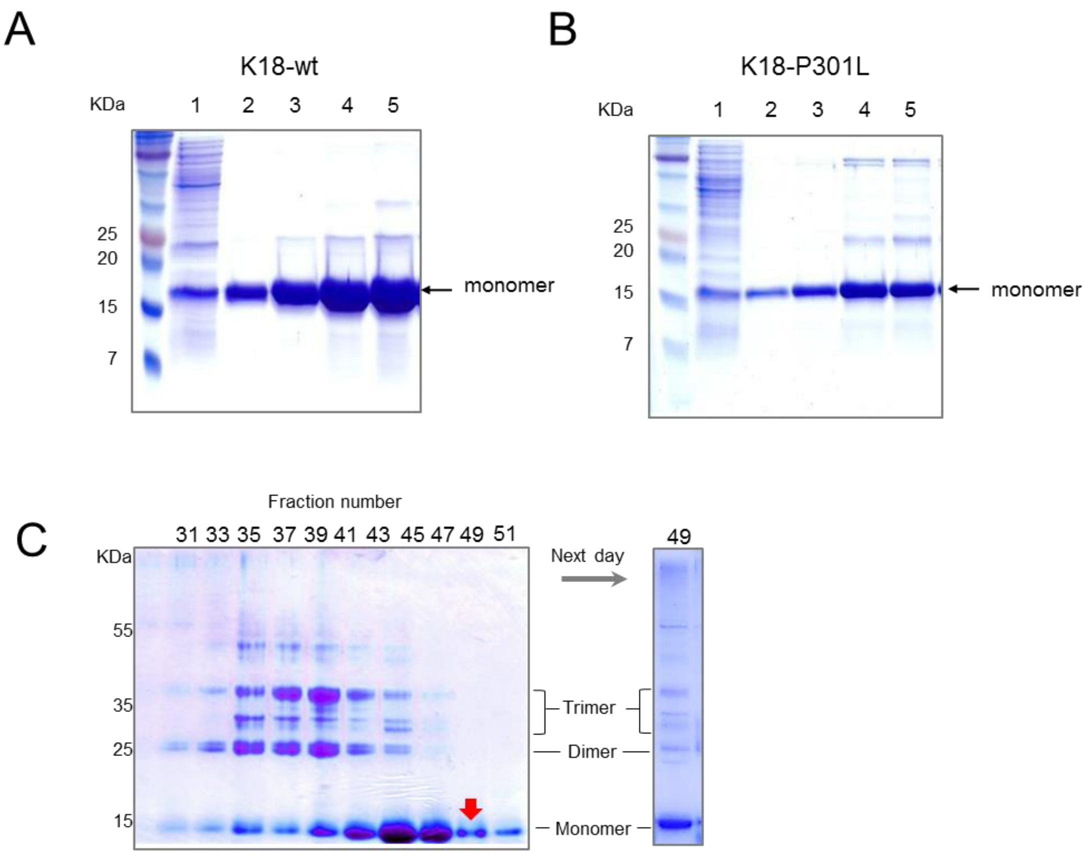

Supplementary Figure 1. Tau assembles spontaneously. Native PAGE analysis shows the preparation of K18-wt (**A**) and K18-P301L (**B**). Lane 1 indicates soluble oligomers after His-tag purification through native PAGE analysis. Lane 2-5 show purified monomers. (**C**) K18-wt was separated as a monomer using FPLC (fraction #49). Next day, the separated monomer reassembled in PBS buffer.

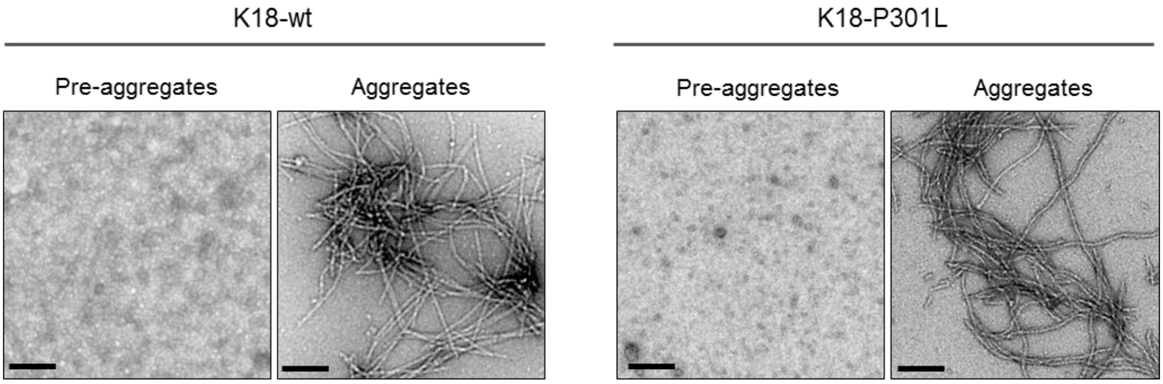

Supplementary Figure 2. Transmission electron microscopy of pre-aggregates and aggregates of K18-wt and K18-P301L. Scale bar = 200 nm

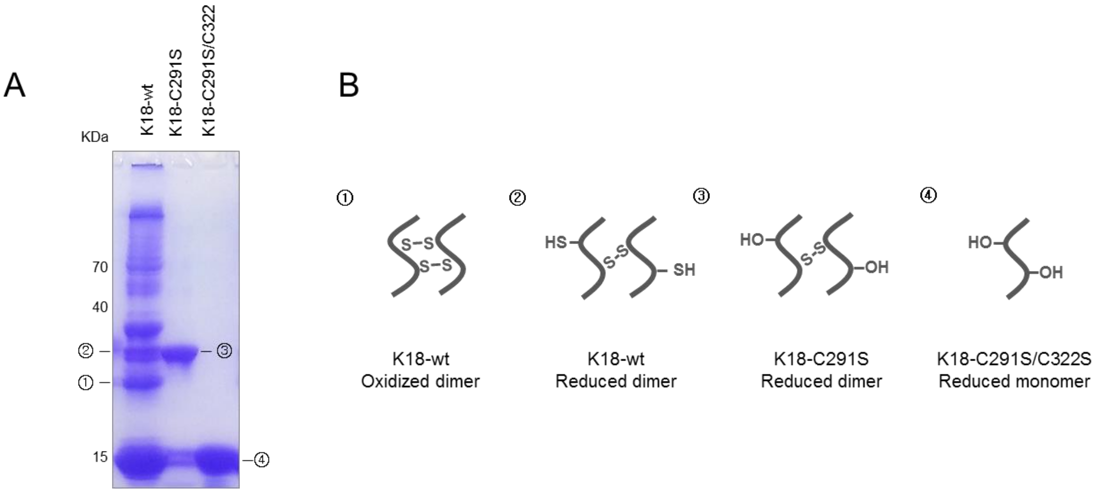

Supplementary Figure 3. Cysteine mutation of tau inhibits oligomerization. (**A**) Non-reducing SDS-PAGE (10-20 %) shows the effect of cysteine mutations in disulfide cross linking for tau oligomerization. (**B**) Illustration of possible disulfide cross-linked dimers of K18-wt and molecular species of monomer or dimer of cysteine mutated-K18 peptides.

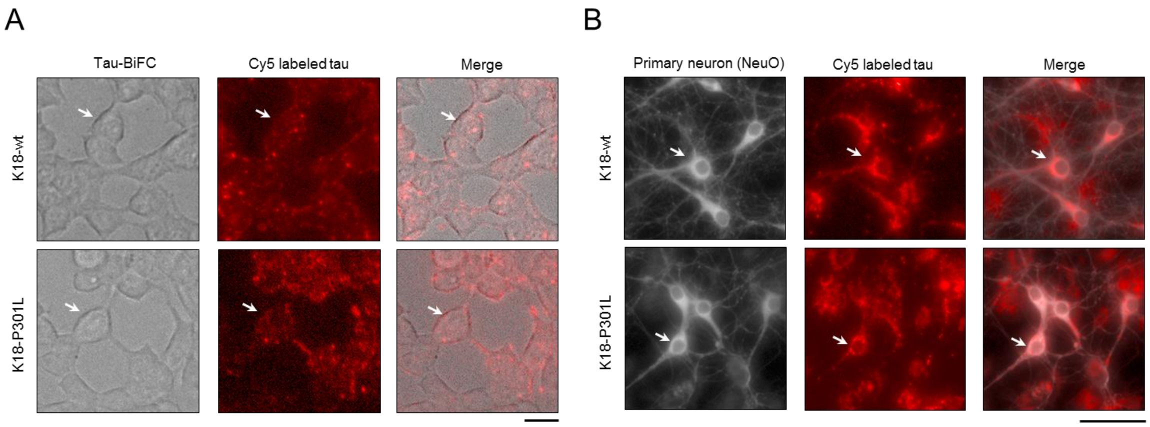

Supplementary Figure 4. Internalization is not a factor leading the prion-like activity. To evaluate the internalization of K18-wt and K18-P301L, the N-terminal amine of K18-wt and K18-P301L were labeled with Cy5. Then, the labeled K18-wt and K18-P301L were treated to tau-BiFC (**A**) or primary neurons (**B**). Fluorescence microscopy images indicate that there was no significant difference on cellular uptake of K18-wt and K18-P301L. Scale bar = 10 μm (**A**) and 50 μm (**B**)

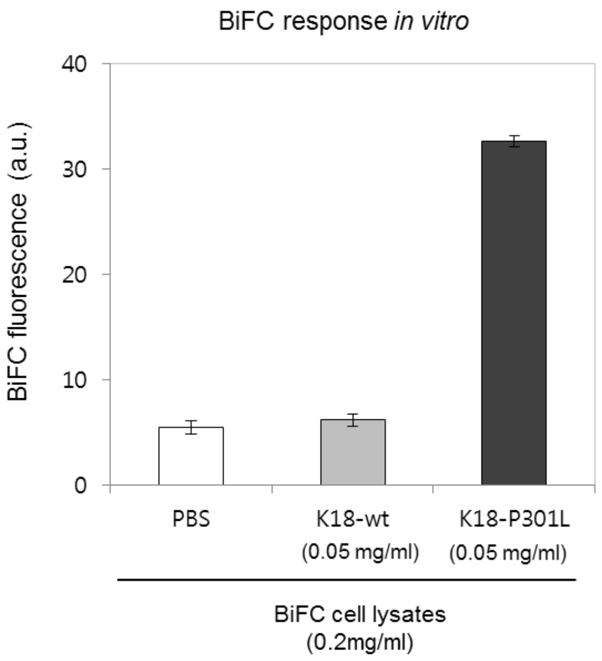

Supplementary Figure 5. Seeding efficiency of K18-P301L for tau aggregation *in vitro*. To test seeding efficiency of K18-wt and K18-P301L, BiFC cell lysates (0.2 mg/ml) was incubated K18-wt or K18-P301L. After 2 days of incubation, increased BiFC response was observed in K18-P301L-incubated cell lysates indicating full-length tau aggregation. (λ_{ex} = 430 nm; λ_{em} =525 nm)

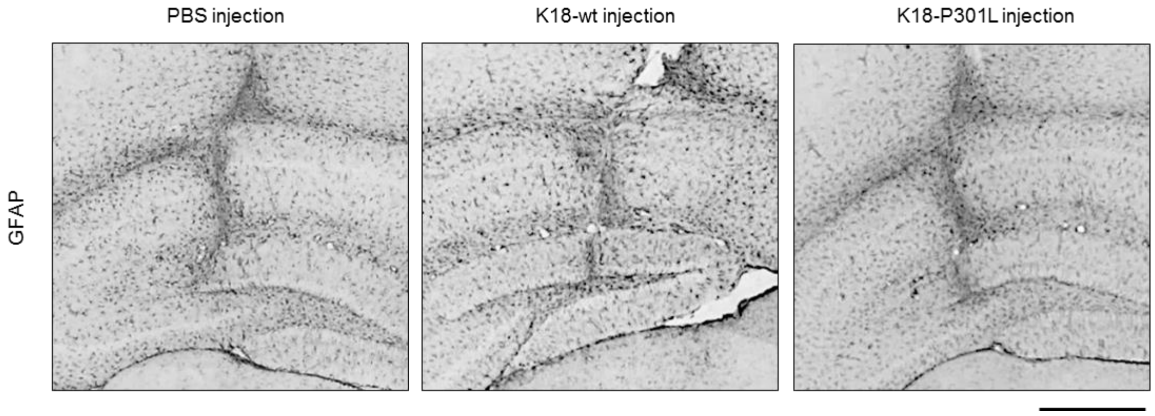

Supplementary Figure 6. Glial fibrillary acidic protein reactivity is induced by tissue damage. Immuno-fluorescence stain with anti-glial fibrillary acidic protein (GFAP) antibody represents reactive astrocyte activation caused by PBS, K18-wt and K18-P301L injection. Scale bar = 500 µm

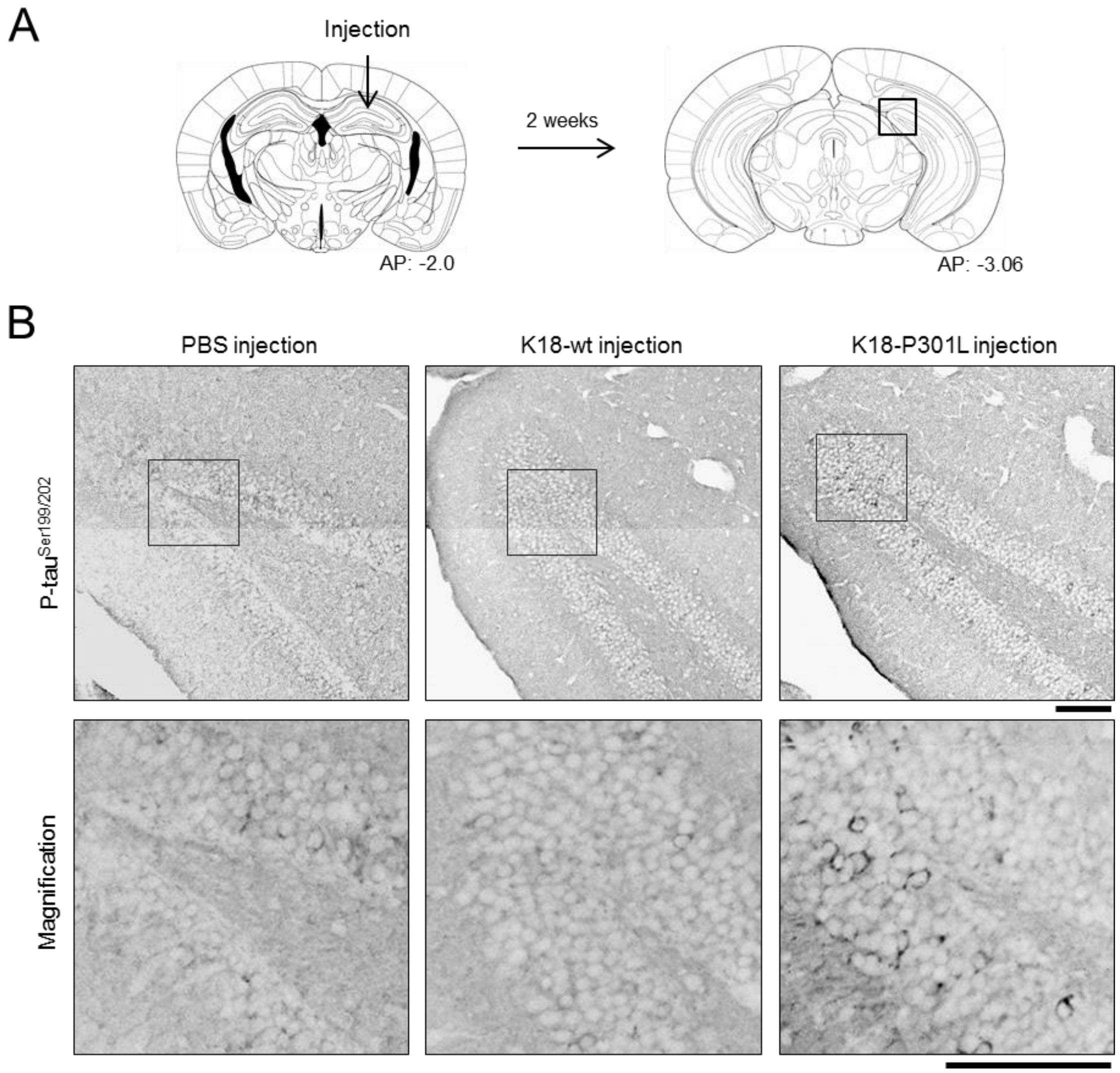

Supplementary Figure 7. Propagation capacity of K18-P301L. (**A**) Brain slices featuring dentate gyrus (AP: -3.06) were selected for immunostaining. The atlas images were from Mouse brain in stereotaxic coordinates[29] (**B**) The representative images of the brain slices stained with anti-phospho tau Ser199/202 antibody. After 2 weeks of injection, only K18-P301L injected mouse brain showed neurons bearing hyperphosphorylated tau. The lower panels are the high-magnification images of the selected regions. Scale bar = 100 μm.

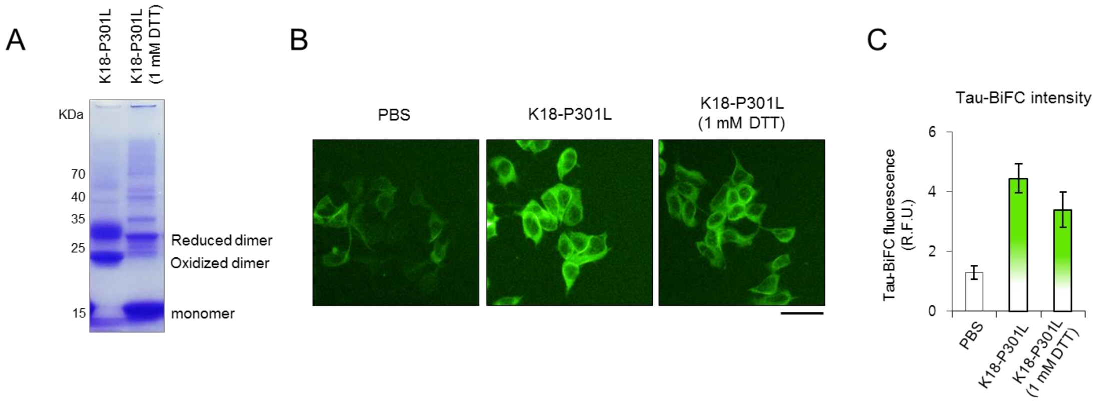

Supplementary Figure 8. Disulfide bridging is important in inducing intracellular tau aggregation. (A) Non-reducing SDS-PAGE shows that K18-P301L dimers were monomerized after high concentration of DTT (1 mM) treatment. (**B**) Tau-BiFC response was lower under reducing condition than that of K18-P301L dimer. Tau-BiFC cells were imaged by using Operetta® High Content Imaging System. Scale bar = 50 µm (**C**) The intensity of BiFC fluorescence was quantified by using Harmony™ software. Error bar indicates S.D. of triplicate experiments.

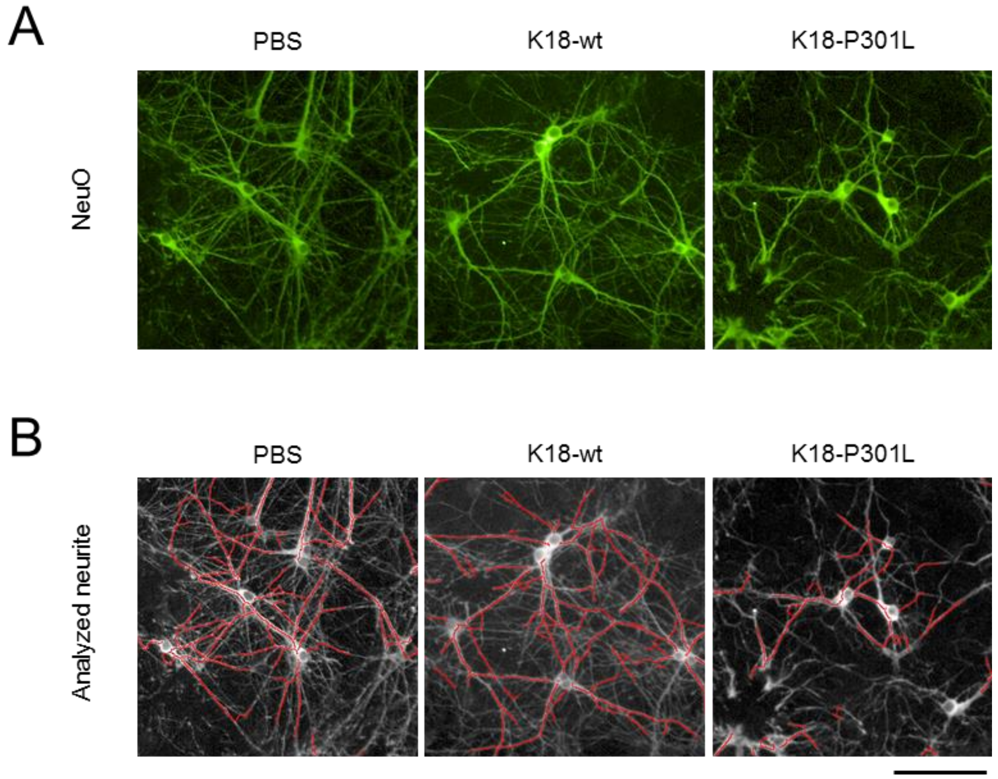

Supplementary Figure 9. Quantification of neurite integrity by NeuO. (A) Fluorescence images of NeuO stained neurons. (**B**) Image analysis for the quantification of neurite length and the number of neurites. Scale bar = 100 µm

Supplementary Methods

Fast protein liquid chromatography (FPLC)

To isolate subspecies of tau, K18 was purified by fast protein liquid chromatography (FPLC). Before the FPLC experiment, K18 was concentrated by Amicon Ultra-15 Centrifugal Filter Units 10K (Millipore). The collected K18 was loaded on Superdex 75 (10/300) column directly. The column was equilibrated with phosphate-buffered saline (PBS, pH 7.4). FPLC was performed at 0.5 mL/min and total volume of eluted fractions was 25 mL.

Transmission electron microscopy (TEM)

Pre-aggregates and aggregates of K18-wt and K18-P301L were analyzed by TEM for assessment of filament formation. Samples were onto carbon-coated copper grids, and then the samples were negatively stained with 2 % uranyl acetate for 60 s. Grids were viewed on a Tecnai F20 transmission electron microscope (FEI, The Netherlands) operating at a voltage of 120 kV.

Fluorescence probe labeling with K18-wt and K18-P301L

K18-wt and K18-P301L (1 mg/ml) were prepared for labeling with florescence probe. Protein solutions were adjusted to the range of 8.0-9.0 using 1 M sodium phosphate buffer (pH 9.0). FNR-648 NHS ester (Bioacts) was dissolved in DMSO to prepare 10 mM stock. After determination the best dye/protein ratio (5:1), protein solutions and FNR-648 NHS ester were mixed and incubated for 2 hrs at RT with shaking. To purify fluorescence labeled with K18-wt and K18-P301L, the reaction mixtures were centrifuged in 10 K

column and the top of the 10 K column was measured at excitation wavelength of 648 nm in Flexstation2 spectrophotometer (Molecular Devices) by the time when the fluorescence signal was disappeared. To check the internalized K18-wt and K18-P301L, the Cy5-labeled proteins (20 μg/ml) were treated to tau-BiFC or primary neurons for 24 hrs. After 24 hrs, cells were washed with growth medium several times and imaged by using Operetta (PerkinElmer™); λex:620-640 nm, λem:650-760

Quantification of Tau-BiFC response in vitro

To quantify tau-BiFC response by K18-wt and K18-P301L *in vitro*, tau-BiFC cell lysates were prepared by using CelLytic M (Sigma) containing protease and phosphatase inhibitor cocktail (Sigma). 0.2 mg/ml of BiFC cell lysates were incubated K18-wt (0.05 mg/ml) or K18-P301L (0.05 mg/ml). 2 days after incubation, 40 µL BiFC cell lysates were transferred to a black 384-well plate and the BiFC fluorescence signal was measured at excitation wavelength of 430 nm in Flexstation2 spectrophotometer (Molecular Devices).

Quantification of neurite integrity by NeuO

For NeuO staining, primary neurons were labeled with 250 nM of NeuO and 2 µg/ml of Hoechst. After 1 hr, primary neurons were imaged by using Operetta (PerkinElmer™); λ_{ex}:460-490 nm, λ_{em}:500-550 for NeuO; λ_{ex}:360-400 nm, λ_{em}:410-480 for Hoechst 33342. For neuron analysis, neurite length and the number of extremities were quantified by Harmony 3.1 software (PerkinElmer™); first, nuclei were detected by Hoechst stain. Second, cell bodies were estimated by extending the nucleus mask. Third, neurites were

detected from the estimated cell body in NeuO stain. Finally, the detected neurites were automatically calculated a set of neurite properties, such as maximal neurite length and the number of extremities.
